# Die neue S3-Leitlinie Schizophrenie (living) 2025

**DOI:** 10.1007/s00115-026-01946-7

**Published:** 2026-02-02

**Authors:** Theresa Halms, Wolfgang Gaebel, Stefan Leucht, Peter Falkai, Tania Lincoln, Andreas Bechdolf, Christoph U. Correll, Stefanie J. Schmidt, Martin Lambert, Thomas Wobrock, Alkomiet Hasan

**Affiliations:** 1 Klinik für Psychiatrie, Psychotherapie und Psychosomatik, Medizinische Fakultät, Universität Augsburg, Geschwister-Schönert-Straße 1, 86156 Augsburg, Deutschland; 2 Kliniken der Heinrich-Heine-Universität, Medizinische Fakultät, LVR-Klinikum Düsseldorf, Düsseldorf, Deutschland; 3 WHO Collaborating Centre on Quality Assurance and Empowerment in Mental Health, DEU-131, LVR-Klinikum Düsseldorf, Düsseldorf, Deutschland; 4 Klinik und Poliklinik für Psychiatrie und Psychotherapie, Klinikum rechts der Isar, Technische Universität München, München, Deutschland; 5 Standort München/Augsburg, Deutsches Zentrum für psychische Gesundheit (DZPG), Augsburg/München, Deutschland; 6 Klinik und Poliklinik für Psychiatrie und Psychotherapie, Klinikum der Universität München, Ludwig-Maximilians-Universität München, München, Deutschland; 7 Klinische Psychologie und Psychotherapie, Fakultät für Psychologie und Bewegungswissenschaft, Universität Hamburg, Hamburg, Deutschland; 8 Kliniken für Psychiatrie, Psychotherapie und Psychosomatik mit FRITZ am Urban & soulspace, Vivantes Klinikum Am Urban und Vivantes Klinikum im Friedrichshain, Berlin, Deutschland; 9 Klinik für Psychiatrie und Psychotherapie, CCM, Charité Universitätsmedizin Berlin, corporate member of Freie Universität Berlin and Humboldt-Universität zu Berlin, Berlin, Deutschland; 10 Standort Berlin/Potsdam, Deutsches Zentrum für Psychische Gesundheit (DZPG), Berlin/Potsdam, Deutschland; 11 Klinik für Psychiatrie, Psychosomatik und Psychotherapie des Kindes- und Jugendalters, CVK, Charité Universitätsmedizin Berlin, corporate member of Freie Universität Berlin and Humboldt-Universität zu Berlin, Berlin, Deutschland; 12 Department of Psychiatry and Molecular Medicine, Donald and Barbara Zucker School of Medicine at Hofstra/Northwell, Hempstead, NY USA; 13 Abteilung für Klinische Psychologie und Psychotherapie, Universität Bern, Bern, Schweiz; 14 Klinik und Poliklinik für Psychiatrie und Psychotherapie, Zentrum für Psychosoziale Medizin, Universitätsklinikum Hamburg-Eppendorf, Hamburg, Deutschland; 15 Zentrum für Seelische Gesundheit, Kreiskliniken Darmstadt-Dieburg, Groß-Umstadt, Deutschland; 16 Klinik für Psychiatrie und Psychotherapie, Universitätsmedizin Göttingen, Göttingen, Deutschland

**Keywords:** Evidence Ecosystem, GRADE, Living Guideline, Evidenzbasierte Medizin, AWMF, Evidence Ecosystem, GRADE, Living guideline, Evidence-based medicine, Association of the Scientific Medical Societies in Germany

## Abstract

**Hintergrund:**

Am 15.10.2025 erschien die neue S3-Leitlinie Schizophrenie (living). Sie wurde erstmals vollständig in das Evidence Ecosystem MAGICapp überführt und wird künftig jährlich aktualisiert.

**Fragestellung:**

Diese narrative Übersichtsarbeit fasst zentrale Neuerungen der Leitlinie 2025 zusammen und erläutert deren Hintergründe.

**Material und Methoden:**

Neue und überarbeitete Empfehlungen sowie Hintergrundtexte aus MAGICapp wurden extrahiert und zusammengeführt.

**Ergebnisse und Diskussion:**

Die Leitlinie ist die erste Leitlinie der Deutschen Gesellschaft für Psychiatrie und Psychotherapie, Psychosomatik und Nervenheilkunde (DGPPN), die vollständig in MAGICapp integriert wurde und als Living Guideline erscheint. Zentrale Änderungen betreffen die Abschwächung der bisherigen antipsychotischen Monotherapieempfehlung, eine deutliche Erweiterung und Präzisierung des Nebenwirkungs- und Sicherheitsmonitorings sowie die Aufwertung evidenzbasierter psychotherapeutischer Verfahren, darunter u. a. das metakognitive Training und die systemische Therapie. Zudem wurden vier neue Empfehlungen verabschiedet (digitale/technikgestützte Interventionen, achtsamkeitsbasierte Verfahren, Acceptance and Commitment Therapy [ACT], traumafokussierte Therapie der posttraumatischen Belastungsstörung [PTBS]) und zwölf gestrichen. Alle Kapitel wurden umfassend überarbeitet.

## Abstract (en)

**Background:**

On 15 October 2025, the revised S3 guideline on schizophrenia (living) was published and transferred to the MAGICapp evidence ecosystem and will be updated annually in the future.

**Objective:**

This narrative review summarizes essential innovations and explains their development.

**Material and methods:**

New and revised recommendations and MAGICapp background information were extracted and summarized.

**Results and conclusion:**

The guideline is now fully integrated into MAGICapp and annually updated. Key changes include a downgrading of the recommendation for antipsychotic monotherapy, a substantial expansion of guidance on adverse effect monitoring and management and a strengthened emphasis on evidence-based psychotherapeutic interventions, such as metacognitive training and systemic therapy. Additionally, 4 new recommendations (digital/technology-assisted interventions, mindfulness-based procedures, acceptance and commitment therapy [ACT], trauma-focussed therapy for post-traumatic stress disorder [PTSD]) were implemented and 12 were removed. All chapters were comprehensively revised.

Die S3-Leitlinie Schizophrenie (living) 2025 bringt grundlegende Änderungen: vollständige Integration in der von der AWMF empfohlenen, webbasierten Plattform zur Entwicklung, Aktualisierung und Veröffentlichung von medizinischen Leitlinien MAGICapp („Making GRADE the Irresistible Choice authoring and publication platform“), konsequente Anwendung der GRADE-Methodik und jährliche Aktualisierung. Vier neue Empfehlungen wurden aufgenommen, zentrale Kapitel – vor allem Pharmakotherapie, Psychotherapie und Nebenwirkungsmanagement – überarbeitet. Der Beitrag erläutert kurz die methodischen Hintergründe und Evidenzbasis und diskutiert mögliche Folgen für die Versorgung.

## Hintergrund

Die Schizophrenie-Leitlinie war 2006 (zweite Version) die erste DGPPN-Leitlinie auf S3-Niveau und liegt seit dem 15.10.2025 in ihrer 4. Version vor. Vorausgegangen war ein umfassender Revisionsprozess der 2019er-Leitlinie unter Einbezug der bis zum 13.06.2025 publizierten Literatur sowie die Überführung in ein Evidence Ecosystem und den Status einer jährlich zu aktualisierenden Living Guideline. Die DGPPN-S3-Leitlinie Demenzen hat eine ähnliche Entwicklung durchlaufen [2], während die Schizophrenie-Leitlinie im G‑BA-Innovationsfonds-Projekt SISYPHOS [3] vollständig in ein digitales Evidence Ecosystem integriert wurde.

Diese Übersicht stellt das Konzept der Living Guidelines und das Evidence Ecosystem MAGICapp vor und fasst die zentralen Neuerungen der Leitlinie Schizophrenie zusammen. Zudem werden Hintergründe veränderter Empfehlungsgrade erläutert. Die Leitliniengruppe hat – mit Ausnahme einzelner Empfehlungen zur repetitiven Transkraniellen Magnetstimulation (rTMS) – alle Empfehlungen der Version von 2019 überprüft und neu konsentiert. Insgesamt wurden vier neue Empfehlungen verabschiedet, zwölf Empfehlungen und vier Statements gestrichen. Die ergänzenden Texte zur Off-Label-Anwendung wurden erweitert, um praktische Orientierung im klinischen Alltag zu geben. Alle Empfehlungen wurden hinsichtlich Off-Label-Gebrauch geprüft und entsprechend gekennzeichnet. Diese Publikation basiert auf der neuen S3-Leitlinie Schizophrenie (living); erforderliche Inhalte wurden unverändert übernommen.

### Infobox

- Leitlinie (Langversion und Kurzversion) sowie Methodenreport sind hier abrufbar: https://register.awmf.org/de/leitlinien/detail/038-009
- Leitlinie in der MAGICapp ist hier abrufbar: https://app.magicapp.org/#/guideline/jlYvkL
- Veröffentlichungsdatum: 15.10.2025, gültig bis: 30.06.2026
- Nächste Living-Leitlinienkonferenz zur Aktualisierung 2026 findet am 08.05.2026 statt

## Methodische Neuerungen in der S3-Leitlinie Schizophrenie

Die wesentlichen methodischen Neuerungen umfassen:

- die Überführung der Leitlinie in das Evidence Ecosystem MAGICapp,
- die Etablierung einer jährlich aktualisierten Living Guideline,
- die Nutzung der GRADE(Grading of Recommendations, Assessment, Development and Evaluation)-Methodik,
- einen strengeren Umgang mit Interessenskonflikten.

Die digitale Plattform MAGICapp wird im AWMF-Regelwerk zur strukturierten Verwaltung und Verbreitung von Living Guidelines empfohlen. MAGICapp, gegründet von Per Olav Vandvik, Linn Brandt, Gordon Guyatt und Thomas Agoritsas, basiert vollständig auf GRADE und ermöglicht daher eine konsistente Evidenzbewertung. GRADE bewertet die verfügbare Evidenz aus einer Outcomeperspektive und beurteilt die Gesamtheit der Evidenz („body of evidence“) für jeden relevanten Endpunkt [1].

Als nächster Schritt wird ein flexibles „Evidence-to-decision“-Framework genutzt, das Kriterien wie Relevanz der Fragestellung, Evidenzqualität, Nutzen-Risiko-Verhältnis, Werte und Präferenzen, Ressourcenverbrauch, Umsetzbarkeit sowie mögliche Ungleichheiten im Gesundheitswesen berücksichtigt [1]. MAGICapp bietet hierzu eine optische Aufbereitung der Evidenz und des Frameworks. Literaturangaben können direkt verlinkt werden, zudem lassen sich praktische Hinweise zu einzelnen Empfehlungen integrieren. Empfehlungen verschiedener Leitlinien können verknüpft und Empfehlungsart, -stärke und Aktualisierungsstand übersichtlich dargestellt werden. Eine Kommentarfunktion ermöglicht kontinuierliche öffentliche Konsultation.

Da sich viele Funktionen erst im digitalen Format erschließen, empfehlen die Autorinnen und Autoren die Nutzung der MAGICapp-Version der Leitlinie. Eine weitere methodische Neuerung betrifft – zusätzlich zu den üblichen AWMF-Regeln – die vollständige Stimmenthaltung der gesamten Schizophrenie-Living-Guideline-Gruppe (Steuerungs- und Expertengruppe) bei allen Konsentierungen, unabhängig von individuellen Interessenskonflikten.

## Die vier De-novo-Empfehlungen

Im Rahmen der Bedarfsanalyse wurden vor der Revision vier Themenbereiche identifiziert, in denen neue Empfehlungen erforderlich waren. Grundlage waren neue Evidenz oder klinischer Bedarf. Folgende vier Empfehlungen wurden erarbeitet und verabschiedet:

- Empfehlung 63 (schwache Empfehlung): „*Menschen mit einer Schizophrenie sollten therapeutisch-begleitete validierte digitale oder technik-gestützte Interventionen (wie AVATAR-Therapie zur Behandlung von auditiven verbalen Halluzinationen (Stimmenhören)) zur Verbesserung der Symptomatik oder Rückfallprävention als Teil eines multimodalen Gesamttherapiekonzeptes angeboten werden*“ (92 % Konsens).
- Empfehlung 77 (starke Empfehlung): „*Menschen mit einer Schizophrenie soll zur Reduktion der Positivsymptomatik eine achtsamkeitsbasierte Therapie angeboten werden*“ (97 % starker Konsens).
- Empfehlung 79 (konsentierte Empfehlung): „*Menschen mit einer Schizophrenie kann eine Acceptance and Commitment Therapy (ACT) angeboten werden*“ (96 % starker Konsens).
- Empfehlung 82 (schwache Empfehlung): „Menschen mit einer Schizophrenie und einer komorbiden PTBS sollte eine trauma-fokussierte Psychotherapie (Prolonged Exposure oder EMDR) zur Reduktion der PTBS Symptomatik angeboten werden“ (100 % starker Konsens).

## Neuerungen im Kapitel Pharmakotherapie

Die antipsychotische Pharmakotherapie bildet gemeinsam mit der Psychotherapie eine zentrale Säule der Behandlung. Zwei Aspekte wurden grundlegend verändert: Zum einen wurde das seit 2006 bestehende Monotherapie-Diktum vor dem Hintergrund neuer Evidenz abgeschwächt, zum anderen das Nebenwirkungskapitel deutlich erweitert (u. a. Management von Gewichtszunahme, Clozapin-assoziierter Myokarditis, neue Vorgaben zum Neutrophilenmonitoring, Pneumonien).

Die frühere starke Empfehlung zur antipsychotischen Monotherapie (Empfehlung 31) wurde von A auf B herabgestuft. Die ursprüngliche Empfehlung beruhte vor allem auf Zulassungsstudien, die nahezu ausschließlich Monotherapien untersuchten. Die neue Bewertung berücksichtigt sowohl die klinische Praxis als auch aktuelle Registerdaten, die Vorteile kombinierter antipsychotischer Behandlungen ohne erhöhte Komplikationsraten zeigen. Randomisierte Studien zu allen relevanten Kombinationsmöglichkeiten wären praktisch nicht durchführbar; Registerdaten bieten daher die nötigen Fallzahlen. Vor diesem Hintergrund wurde die Empfehlung zur Monotherapie abgeschwächt. Betont wurde die Bedeutung, die verschiedenen Nebenwirkungen der antipsychotischen Therapie zu überwachen und auf solche zu reagieren.

Parallel wurden die Clozapin-Empfehlungen (44a–c) überarbeitet. Clozapin soll weiterhin als Monotherapie bei Therapieresistenz eingesetzt werden (starke Empfehlung). Ist Clozapin nicht möglich, nicht verträglich oder nicht gewünscht, kann eine Kombination aus maximal zwei Antipsychotika angeboten werden.

Weitere Neuerungen im Kapitel Pharmakotherapie:

- Die Bedeutung des therapeutischen Drug-Monitorings (TDM) bei Clozapin wurde gestärkt; der Empfehlungsgrad wurde von B auf A angehoben.
- Cariprazin wurde zur Behandlung der Negativsymptomatik ergänzt, Olanzapin gestrichen.
- Im Nebenwirkungsmanagement wurden die Hintergrundtexte umfassend überarbeitet. Die Schwelle für eine therapeutische Reaktion auf Gewichtszunahme wurde von 7 % auf 3 % gesenkt (Empfehlung 53).
- Die Empfehlung zur Off-Label-Gabe von Metformin bei > 3 % Gewichtszunahme wurde nach GRADE-Bewertung als starke Empfehlung beibehalten. Topiramat wurde aufgrund schwächerer Evidenz und eines Rote-Hand-Briefs gestrichen.
- Eine präventive Metformin-Gabe, international in bestimmten Konstellationen und bei Anwendung von Clozapin oder Olanzapin stets empfohlen, wurde in Deutschland aufgrund rechtlicher Rahmenbedingungen (keine präventive Off-Label-Behandlung) nicht übernommen; vor diesem Hintergrund wurde die 3‑%-Grenze festgelegt.
- Glucagon-like Peptide-1(GLP-1)-Rezeptoragonisten wurden diskutiert; wegen noch fehlender Evidenz (für Ende 2025 sind mehrere Publikationen hier zu erwarten) und fehlender Erstattungsmöglichkeiten zulasten der gesetzlichen Krankenversicherung in dieser Indikation wird eine Entscheidung auf zukünftige Zyklen verschoben.
- Neue Vorgaben zur Clozapin-Überwachung (u. a. aktualisiertes Blutbildmonitoring, Myokarditisempfehlungen) wurden ergänzt. Das Nebenwirkungskapitel wurde um weitere Inhalte wie Prolaktinerhöhung und Pneumonie erweitert.

## Neuerungen im Kapitel Psychotherapie

Alle oben dargestellten De-novo-Empfehlungen sind dem Kapitel Psychotherapie zuzuordnen. Weitere Änderungen führten überwiegend zu einer Aufwertung oder Differenzierung von Empfehlungsgraden; nur in einem Bereich wurde der Grad reduziert.

- Psychoedukation: Die seit 2006 hoch bewertete Empfehlung wurde neu bewertet und zweigeteilt. Psychoedukation unter Einbeziehung von Angehörigen (57a) weist eine stärkere Evidenz auf als die bifokale Psychoedukation (57b). Beide Formen bleiben klar empfohlen und wichtige Bestandteile der Behandlung; lediglich die Empfehlungsgrade wurden differenziert.
- Metakognitives Training: Der Empfehlungsgrad wurde aufgrund neuer Evidenz von B auf A angehoben (Empfehlung 64).
- Systemische Therapie: 2019 noch mit dem niedrigsten Empfehlungsgrad bewertet, wurde die Evidenz neu geprüft, um Inkonsistenzen zu anderen Empfehlungen und zu früheren Bewertungen aufzulösen. Unter Einbezug aktueller Analysen wurde der Empfehlungsgrad der systemischen Therapie (67) von 0 auf B erhöht.
- Kognitive Remediation: Die Empfehlungen 73 und 74 wurden inhaltlich konkretisiert; der Empfehlungsgrad A bleibt bestehen.

## Neuerungen in den psychosozialen Therapien

Zum Zeitpunkt der Veröffentlichung der neuen S3-Leitlinie Schizophrenie lag die S3-Leitlinie Psychosoziale Therapien bei schweren psychischen Erkrankungen noch nicht vor; beide Leitliniengruppen stimmten sich jedoch ab, um Widersprüche zu vermeiden. Auf Basis neuer Evidenz wurde der Empfehlungsgrad zur Ergotherapie (76) von 0 auf B angehoben. Der Empfehlungsgrad für Bewegungsinterventionen (80) wurde von B auf A (starke Empfehlung) erhöht.

## Neuerungen im Bereich Kinder- und Jugendpsychiatrie und -psychotherapie

In diesem Kapitel wurden keine neuen Evidenzen identifiziert, jedoch die vorhandene Evidenz neu bewertet. Grundlage war eine Bedarfsanalyse aus Sicht der Kinder- und Jugendpsychiatrie und -psychotherapie. Die Darstellung der verfügbaren Antipsychotika („on-label“) sowie der Präparate mit Wirksamkeitsnachweis in dieser Population („off-label“) wurde überprüft und die Listen entsprechend überarbeitet. Zudem wurde die Clozapin-Empfehlung an die für Erwachsene angeglichen, sodass der Empfehlungsgrad für Menschen unter 18 Jahren (114) von B auf A angehoben wurde.

## Neuerungen im Bereich erhöhtes Psychoserisiko

Im Jahr 2019 enthielt die Leitlinie noch eine Empfehlung zur Behandlung von Menschen mit erhöhtem Psychoserisiko. Diese wurde nun in drei Empfehlungen (128a–c) aufgeteilt, und der Empfehlungsgrad für eine Kognitive Verhaltenstherapie (KVT) zur Reduktion oder Verzögerung eines Übergangs in eine Schizophrenie wurde von A auf B abgesenkt. Hintergrund ist eine veränderte Evidenzlage: Während frühere paarweise Metaanalysen positive Effekte zeigten, fand eine aktuelle Netzwerkmetaanalyse keinen signifikanten Zusatznutzen. Die Leitliniengruppe diskutierte methodische Limitationen dieser Analyse, darunter geringe Studienzahl, geringe Power und breite Konfidenzintervalle bei großer Heterogenität. Da in diesem klinischen Stadium zudem wenige therapeutische Alternativen bestehen, wurde die Empfehlung beibehalten, jedoch im Grad abgeschwächt.

Die Empfehlungen wurden wie folgt formuliert (alle mit 100 % starkem Konsens):

- *128a:* Menschen mit erhöhtem Psychoserisiko sollte eine KVT zur Reduktion oder Verzögerung eines Übergangs in eine Psychose angeboten werden.
- *128b:* Eine Behandlung mit Antipsychotika zu diesem Zweck soll nicht angeboten werden.
- *128c:* Wenn KVT nicht ausreicht und attenuierte oder kurze psychotische Symptome zunehmen, können Antipsychotika der zweiten Generation in geringer Dosierung nach sorgfältiger Risiko-Nutzen-Abwägung vorübergehend zusätzlich zur Symptomreduktion eingesetzt werden („off-label“).

## Qualitätsindikatoren

Die Qualitätsindikatoren wurden nicht neu entwickelt, sondern aus der Leitlinie von 2019 übernommen, da die Evidenzprüfung keine neuen indikatorgeeigneten Bereiche für diese Zielpopulation ergab. Die Konsensusgruppe entschied daher, das bestehende Set unverändert zu belassen. Das zugehörige Statement 4 wurde als nicht evidenzbasierter Expertenkonsens überarbeitet. Mit starkem Konsens wurde festgehalten, dass leitlinienbasierte Qualitätsindikatoren für die Schizophreniebehandlung als Instrument des Qualitätsmanagements realisierbar sind.

## Implementierung

Im Rahmen der Überführung in die MAGICapp wurde im G‑BA-Innovationsfonds-Projekt SISYPHOS (siehe Details im zitierten Ergebnisbericht) der Implementierungsstand der S3-Leitlinie Schizophrenie untersucht. Dabei zeigte sich, dass künftig berufsgruppenspezifische Maßnahmen notwendig sind, um eine breite Anwendung der Leitlinie in allen Professionen zu erreichen. Konkret waren Kenntnisse bezüglich der Leitlinie an sich und die Überzeugung der Notwendigkeit der Anwendung vor allem bei Pflegefachpersonen und Mitarbeitenden in den Komplementärtherapien nicht ausreichend. Das Projekt zeigte zudem, dass digitale zweiwöchentliche Frage-und-Antwort-Formate sowie Kurzschulungen das Leitlinienwissen deutlich verbessern. Für die Nutzung der MAGICapp wurde eine gute Anwenderfreundlichkeit und ein schneller Wissenszuwachs festgestellt. Vor diesem Hintergrund ist im Rahmen der Living-Revisionen die Entwicklung eines Onlineschulungskonzepts geplant.

## Schlussfolgerung

Die Überführung der S3-Leitlinie Schizophrenie in ein Evidence Ecosystem und in eine Living Guideline war ein großer, aber notwendiger Schritt. Angesichts des raschen Wissenszuwachses ist es kaum noch vertretbar, Leitlinien über mehrere Jahre unverändert zu lassen. Daher wird die neue Leitlinie jährlich aktualisiert und enthält zusätzlich ein Kapitel zu Neuentwicklungen, vor allem in der Pharmakotherapie (8.22). In der aktuellen Version wurden vier De-novo-Empfehlungen aufgenommen. Künftig wird vor jeder Revision eine Bedarfsanalyse den konkreten Anpassungsbedarf bestimmen.

Die digitale MAGICapp-Version ist für viele Nutzerinnen und Nutzer neu, bietet jedoch klare Vorteile: umfassende Suchfunktion, Vernetzung und Visualisierung der Evidenz, Darstellung des „Evidence-to-decision“-Frameworks sowie praxisnahe Zusatzinformationen. Die vollständige Überführung erforderte eine komplette Neustrukturierung, die im G‑BA-Projekt SISYPHOS umgesetzt wurde. Dadurch ist die PDF-Version weniger benutzerfreundlich; die Nutzung der digitalen Version wird ausdrücklich empfohlen. Gleichzeitig bringt die laufende Weiterentwicklung der MAGICapp auch technische Herausforderungen mit sich.

## Fazit für die Praxis

- Die S3-Leitlinie Schizophrenie liegt nun als jährlich aktualisierte Living Guideline in MAGICapp vor; die digitale Version bietet klare Evidenzgrade, Off-Label-Hinweise und praxisnahe Zusatzinformationen.
- Zentrale inhaltliche Neuerungen betreffen die Pharmakotherapie: Das langjährige Monotherapie-Diktum wurde vor dem Hintergrund neuer Evidenz abgeschwächt und besser in den Kontext der Behandlungsstufen gesetzt, Nebenwirkungs- und Sicherheitsmonitoring (u. a. Gewichtszunahme, Clozapin-assoziierte Risiken, Blutbildkontrollen) deutlich erweitert und präzisiert.
- Gewichtszunahme erfordert ab 3 % eine Intervention; Metformin bleibt bei > 3 % stark empfohlen.
- Psychoedukation, kognitive Remediation, systemische Therapie, Bewegungsinterventionen und Ergotherapie wurden in ihrer Evidenz gestärkt und sollten regelhaft angeboten werden.
- Vier neue Empfehlungen wurden verabschiedet: digitale/technikgestützte Interventionen, achtsamkeitsbasierte Verfahren, ACT sowie traumafokussierte PTBS-Psychotherapie.
- Berufsgruppenspezifische Implementierungsstrategien und digitale Schulungsformate unterstützen die praktische Anwendung der Leitlinie.

## References

[1] Alonso-Coello P, Schünemann HJ, Moberg J et al (2016) GRADE Evidence to Decision (EtD) frameworks: a systematic and transparent approach to making well informed healthcare choices. 1: Introduction. BMJ (Clinical research ed.) 353:i2016 27353417 10.1136/bmj.i2016

[2] AWMF (2025) AWMF Leitlinienregister - S3 Leitlinie Demenzen (Living Guideline). https://register.awmf.org/de/leitlinien/detail/038-013. Accessed 13.11.2025

[3] SISYPHOS – Strukturierte Implementierung digitaler, systematisch aktualisierter Leitlinienempfehlungen zur optimierten Therapeutenadhärenz bei Schizophrenie - G‑BA Innovationsfonds (2025) SISYPHOS – Strukturierte Implementierung digitaler, systematisch aktualisierter Leitlinienempfehlungen zur optimierten Therapeutenadhärenz bei Schizophrenie - G‑BA Innovationsfonds. https://innovationsfonds.g-ba.de/projekte/sisyphos.404. Accessed 13.11.2025

